# Mesoglycan: Clinical Evidences for Use in Vascular Diseases

**DOI:** 10.1155/2010/390643

**Published:** 2010-08-31

**Authors:** Antonella Tufano, Claudia Arturo, Ernesto Cimino, Matteo Nicola Dario Di Minno, Mirko Di Capua, Anna Maria Cerbone, Giovanni Di Minno

**Affiliations:** Regional Reference Centre for Coagulation Disorders, Department of Clinical and Experimental Medicine, AOU “Federico II” Naples 80131, Italy

## Abstract

Vascular glycosaminoglycans (GAG) are essential components of the endothelium and vessel wall and have been shown to be involved in several biologic functions. Mesoglycan, a natural GAG preparation, is a polysaccharide complex rich in sulphur radicals with strong negative electric charge. It is extracted from porcine intestinal mucosa and is composed of heparan sulfate, dermatan sulfate, electrophoretically slow-moving heparin, and variable and minimal quantities of chondroitin sulfate. Data on antithrombotic and profibrinolytic activities of the drug show that mesoglycan, although not indicated in the treatment of acute arterial or venous thrombosis because of the low antithrombotic effect, may be useful in the management of vascular diseases, when combined with antithrombotics in the case of disease of cerebral vasculature, and with antithrombotics and vasodilator drugs in the case of chronic peripheral arterial disease. The protective effect of mesoglycan in patients with venous thrombosis and the absence of side effects, support the use of GAG in patients with chronic venous insufficiency and persistent venous ulcers, in association with compression therapy (zinc bandages, multiple layer bandages, etc.), elastic compression stockings, and local care, and in the prevention of recurrences in patients with previous DVT following the standard course of oral anticoagulation treatment.

## 1. Introduction

Vascular glycosaminoglycans (GAG) are essential components of the endothelium and vessel wall and have been shown to be involved in several biologic functions. Mesoglycan, a natural GAG preparation, is a polysaccharide complex, rich in sulphur radicals with strong negative electric charge. It is extracted from porcine intestinal mucosa and is composed of heparan sulfate (typical content 52%), dermatan sulfate (35%), electrophoretically slow-moving heparin (8%), and variable and minimal quantities of chondroitin sulfate (5%) [1]. Heparan and dermatan sulphate are thrombin inhibitors acting through complementary pathways, antithrombin (AT), and heparin cofactor II, respectively [1]. Heparan sulphate also inhibits activated factor X (FXa). 

In animal models, heparan sulfate and dermatan sulfate have been shown to display antithrombotic and pro-fibrinolytic properties to prevent atherosclerotic lesions and to regulate the selective permeability at the microcirculatory level [2–6].

Mesoglycan is reported to have several favorable actions on the fibrinolytic system, on macrorheologic and microrheologic parameters, and to restore the electronegativity of the vascular endothelium in case of damage [1, 7, 8]. Mesoglycan has a relevant profibrinolytic activity, after oral administration. This pharmacological activity of mesoglycan could possibly involve the liberation of a certain amount of tissue plasminogen activator (tPa) [1]. Moreover, in patients with vascular atherosclerotic disease and with diabetes, mesoglycan is responsible for an improvement in the dynamic properties of red cell membrane (increased membrane fluidity) [7]. This improvement in the erythrocyte membrane fluidity may be related to the variation in the red cell membrane permeability and to the readjustment of the surface electric charges, mediated by direct or indirect interactions of the administered GAG with the erythrocyte membrane [7].

Mesoglycan and another heparin-like substance, sulodexide, potentiate the mitogenic activity of fibroblast growth factors (FGFs) and protect them from heat denaturation and enzymatic degradation [9]. Mesoglycan seems effective in restoring defective fibrinolysis in patients affected by cutaneous necrotizing venulitis [10], suggesting that in inflammatory vasculitis, characterized by a reduced cutaneous fibrinolysis (reduced release of tPA from the vascular endothelium), the use of a fibrinolytic agent should be considered.

## 2. Clinical Studies

### 2.1. Atherosclerosis and Diabetes Mellitus

The pro-fibrinolytic activity of orally administered mesoglycan has been evaluated in 18 patients affected by impaired plasma fibrinolytic activity [1]. The decreased fibrinolytic activity in the patients studied was due to generalized atherosclerotic vascular disease or to diabetes mellitus. Mesoglycan was administered by a single dose of 24, 48, or 72 mg on 1 day and by repeated doses of 48 mg twice a day, for 9 consecutive days. After the single administration, all the fibrinolytic parameters were significantly and positively influenced by an order of magnitude and a duration of effects proportional to the dose employed. After the repeated administration, a constant and reproducible activation of the fibrinolytic system was observed without any interference with hemocoagulative parameters [1]. The results of this study showed that mesoglycan has a relevant pro-fibrinolytic activity in man after oral administration. The authors supposed that pharmacological activity of mesoglycan could possibly involve the liberation of a certain amount of plasminogen tissue activator [1].

Caimi et al. showed in 10 patients with vascular atherosclerotic disease and in 15 patients with vascular atherosclerotic disease and with non-insulin-dependent diabetes mellitus that mesoglycan, at doses of 100 mg orally twice daily for 30 days, was responsible for an improvement in the dynamic properties of red-cell membrane (increased membrane fluidity) [7]. This improvement in the erythrocyte membrane fluidity may be related to the variation in the red-cell membrane permeability and to the readjustment of the surface electric charges mediated by direct or indirect interactions of the administered GAG with the erythrocyte membrane [7].

### 2.2. Arterial Disease

#### 2.2.1. Cerebrovascular Disease (Table 1)

Based on the evidence that high plasma fibrinogen (FBR) levels are risk factors for ischemic stroke and myocardial infarction (MI) [11–16], Orefice et al. [12] evaluated the effects of mesoglycan on some parameters of the fibrinolytic system in patients with ischemic cerebrovascular disease of an atherosclerotic nature and the side effects of a long-term treatment with this drug. Thirty patients who had previously had an athero-thrombotic stroke, 15 men and 15 women, mean-aged 67 years, received mesoglycan 50 mg twice daily, for 3 months, to evaluate the effect of the drug on plasma FBR levels, AT concentration, and other coagulative parameters. Patients had the last ischemic acute event 1–3 months before. The coagulative parameters were evaluated at baseline 4 hours after the morning dose of mesoglycan and after 7, 30 and 90 days of therapy. After 7 days of treatment, mesoglycan significantly decreased plasma FBR levels (*P *< .05). This result was confirmed after 30 and 90 days (*P *<  .005, and *P *<  .001, resp.). No statistically changes in AT levels, prothrombin time (PT), partial thromboplastin time (PTT), or platelet count were observed with respect to baseline values [12]. 

This drug's effect is poorly understood, it may be correlated in some way to a protective action on the endothelial cells, possibly maintaining the physiologic electronegativity of the endothelial surface. The authors concluded that mesoglycan is a safe and effective drug for reducing plasma FBR levels without interfering with other coagulative parameters [12].

In a randomized controlled trial [11], patients were randomized into 2 groups: one group (*n* = 28; 14 men, 14 women) receiving mesoglycan (50 mg twice daily) and the other group (*n* = 18; 12 men, 6 women) ticlopidine (250 mg twice daily). Criteria for patients enrollement were a baseline FBR levels ≥ 350 mg/dL and age > 40 years. The time of stroke onset ranged from 2 to 3 months before patients entered the study. After 2 months of treatment, the authors found a statistically significant reduction of both functional (*P* =  .01) and immunologic (*P* <  .05) plasma FBR levels in the group receiving mesoglycan; in the patients receiving ticlopidine, these reductions were more significant (*P* <  .01 for both). However, the effect was transient; it disappeared 15 days after the end of treatment. The study showed that mesoglycan may be an effective drugs for reducing plasma FBR levels without interfering with other coagulative parameters and without serious side effects. The results of this study confirm the data of previous reports showing the decrease of plasma FBR levels with some drugs (ticlopidine and fibrates) [17]. Other studies comparing mesoglican and ticlopidine or other tienopiridines in arterial thromboses are lacking in the literature, so these results are not adequately confirmed and validated.

Vecchio et al. evaluated the hematochemical and hemorheologic effects of mesoglycan, administered by the intramuscular route to patients with a recent episode of cerebral ischemia [13]. A total of twenty patients (13 males and 7 females), between the ages of 45 and 75, under observation for a cerebral ischemic episode occurring at least 2 months prior to enrollement were treated with intramuscular mesoglycan (30 mg, twice daily) for 15 days. Blood samples were taken prior to and at the end of treatment to measure the investigated parameters. Following mesoglycan treatment, the authors observed a statistically significant decrease in FBR plasma concentration, total cholesterol and triglycerides, while HDL cholesterol was found to increase. In addition, erythrocytes filterability improved at the end of treatment. No changes were observed in coagulation parameters such as PT, PTT, or AT. In this study, a 15-days treatment of intramuscular mesoglycan in patients recovering from a cerebral ischemic episode produces significant changes in FBR and lipid plasma levels with no apparent anticoagulant effect [13]. The results of the present study further support the concept that mesoglycan reduces plasma FBR levels without altering coagulative parameters.

The mesoglycan efficacy has also been evaluated in chronic cerebrovascular disease, in a study [14] conducted on a group of 30 patients (19 males, 11 females; mean-aged ± SD = 63.6 ± 8.7), with a history of transient ischemic attacks (TIA) (30%) or ischemic strokes (70%) within the 3 months before the treatment. The patients were treated with mesoglycan (50 mg, twice a day) over 3 months. The efficacy of the drug in chronic treatment of cerebrovascular disease was evaluated at basal time and after 30, 60, and 90 days by clinical examination using neurological and neuropsychological rating scales. An improvement in mnesic performances, mood, and autonomy in daily living activity and a reduction in emotional lability was demonstrated by the decrease of the rating scale mean scores. Instability at Romberg test, primitive reflexes, and weakness were the most often reversible neurological signs. This could be ascribed to the effect of the drug on hemoreological conditions [14].

Recently, it has been showed that heparan sulphate proteoglycans are the main antigenic components of cholinergic synaptic vesicles, and may help to maintain the vesicles membrane integrity during multiple cycles of secretion and endocytosis [14].

Interestingly, Forconi et al. [15] compared the efficacy and tolerability of mesoglycan and acetylsalicylic acid (ASA) in the prevention of vascular events after cerebral ischemic events in a large multicenter clinical trial. In 48 Italian clinical centers, 1,398 patients with a history of athero-thrombotic stroke one or more episodes of TIA, reversible ischemic neurological deficit (RIND), or minor stroke with the last episode occurring 14–90 days before admission were randomized to mesoglycan 30 mg i.m. twice daily for two weeks, then 100 mg/day orally (*n* = 701) or ASA 300 mg once daily (*n* = 697) [15]. Primary outcome events were considered: reversible ischemic neurologic deficits, stroke, acute MI, or vascular death. At the end of followup period (median duration 18 months), the number of primary outcome events observed in the two treatment groups was similar (125 in the ASA group, 129 in the mesoglycan group); major stroke was observed in 26 patients of the ASA group and in 26 patients of the mesoglycan group; and deaths from vascular causes were 37 and 32, respectively. Log-rank statistics failed to evidence significant differences between treatment groups. On the other hand, a significantly (*P* <  .01) higher incidence of side effects was reported in the ASA group [15].

These data, and particularly the absence of side effects, suggest that mesoglycan can be safely used in the management of ischemic stroke in patients treated with standard antiplatelet regimens, and additional and larger studies are required to confirm this hypothesis.

#### 2.2.2. Peripheral Obstructive Arterial Disease [18–20] (Table 2)

Andreozzi et al. [18] evaluated the use of mesoglycan in patients with peripheral obstructive arterial disease (POAD). Ten patients with PAOD in stages I and II according to Leriche-Fontaine were treated with mesoglycan sulfate (60 mg daily for twenty days) to evaluate the effects of the drug on the elastic modulus of the arterial wall. The wall elasticity was deduced from some Doppler velocitographic indices: arterial dynamics index (ADI), resistance index (RI), perfusion pressure index (PPI), and tibial distensibility index (TDI) from the analysis of systolic, protodiastolic, and end-diastolic velocity variations and from computerized analysis of the Doppler sound spectrum [18]. The treatment with mesoglycan sulfate induced: (1) a reduction of mean RI and PPI, which expresses a reduction of the peripheral resistance on the arteriolo-capillary side and, thus, a better endothelial function; (2) a reduction in the worsening of the mean percent variations of the systolic window induced by ischemia, which is a sign of a more homogeneous flow of the blood particles inside the vessel and of a better wall responsiveness; and (3) an improvement of the wall response to the vasodilator stimulus [18]. These results, obtained in patients in a very early stage, should be ascribed to the improvement of arteriolar reactivity mediated via the improvement of the elastic modulus.

This study suggested a role of mesoglycan in the therapy of arterial diseases, primarily in the treatment of the initial stages of atherosclerotic, hypertensive, or diabetic arterial diseases, wherein reduced elasticity of arterial wall is one of the earliest functional impairments [18].

Nakashima et al. [21] described 2 patients with chronic arterial occlusion in whom the intravenous (IV) administration of mesoglycan was markedly effective. One patient suffered from thromboembolic episodes of the left hand, due to a mural thrombus formed in an aortic aneurysm, and the other had peripheral circulatory impairment related to a collagen disease (periarteritis nodosa cutanea). In these patients, the oral administration of anticoagulants and antiplatelet agents in combination with IV infusion of prostaglandin E_1_ (PGE1) was not adequately effective. However, the addition of IV injection of mesoglycan (30 mg twice daily) resulted in a marked improvement in the signs and symptoms of both patients [21], suggesting that mesoglycan may be useful for the treatment of chronic arterial occlusion when combined with antithrombotic and vasodilator drugs.

GAG administered IV seem to exert an anticoagulant effects by: inhibiting activated factor X (Xa), activating fibrinolytic agents, and inhibiting platelet aggregation [21, 22].

The effect of mesoglycan was investigated in patients with acute episodes of relative lower limb ischemia (Stage IIb according to Leriche-Fontaine classification) [19]. Mesoglycan was administered according to the following schedule: a 10-days period of intravenous mesoglycan (90 mg/day), given in day-hospital regimen, followed by a 20-days period of oral mesoglycan (100 mg/day). The treatment schedule was repeated for two months and then patient continued with oral mesoglycan. Thirty-six patients were followed for a mean period of 12 months. From February 1995, thirty-six patients, 24 males and 12 females, aged between 45 and 83 years (mean ±  SD: 69.8 ± 7.5) with acute relative lower limb ischemia were enrolled. At baseline, the diagnosis was Fontaine's IIb stage (walking distance < or = 200 m) in all patients, 17 patients presenting walking distance < 100 m. After 3 and 6 months of mesoglycan treatment a significant improvement of symptoms and signs was observed in all patients but one. At the end of the 6-months period, 29 patients (81% of the study population) became to a Fontaine's IIa degree, with a significant increase in walking distance (in 70% three times their basal value) and improvement of symptoms and recovery time. After treatment, Winsor Index was not significantly modified. Similar results were obtained after 12 months of followup. During the study period only one patient included in the trial needed surgical revascularisation. The administration of mesoglycan was well tolerated, with only minor complaints in two patients (one case of headache and one of diarrhea). During the intravenous administration of mesoglycan most patients (81%) presented values of aPTT 2.0 times control, which returned to normal values at the end of the administration. This results showed that in patients with acute episodes of relative lower limb ischemia, mesoglycan (administered according to the described protocol) was an effective and safe agent able to improve symptoms (walking distance, pain, and leg appearance) and to possibly delay the need of surgical interventions.

Nenci et al. [20] studied the effect of treatment with mesoglycan on the walking capacity of patients with stage II peripheral arterial disease. Nondiabetic outpatients with intermittent claudication, duplex ultrasound evidence of peripheral atherosclerosis, ankle/arm index <0.80, systolic ankle pressure >50 mmHg, and absolute walking distances (AWD) between 100 and 300 m (standardized treadmill test) were eligible. After a 5 week run-in on single-blind placebo, 242 patients were randomized to double-blind treatment with mesoglycan, 30 mg/day intramuscularly for 3 weeks followed by 100 mg daily orally, for 20 weeks, or matching placebo. All patients received low-doses aspirin and lifestyle instructions. Clinical response was defined as an AWD increase at Week 23 > 50% over baseline. Health-related quality of life and ischemic events were assessed as secondary efficacy variables [22]. Patients achieving clinical response were 59/118 with mesoglycan (50%) and 31/119 with placebo (26.1%; *P *<  .001). Geometric mean AWD increased from 192 m to 298 m with mesoglycan, and from 192 m to 238 m with placebo (*P* <  .001). Pain-free walking distance showed a nonsignificant increase with mesoglycan (*P* =  .057). Changes in quality of life scores were in favor of mesoglycan. The rate of ischemic events was 1/120 on mesoglycan and 6/122 on placebo (*P*  =  .053). The rate of non ischemic adverse events leading to treatment discontinuation was 7/120 and 4/122, respectively. In conclusion, treatment of intermittent claudication with mesoglycan increased the proportion of patients achieving a clinical interesting (>50%) improvement in maximal walking capacity. The treatment was well-tolerated, even in conjunction with antiplatelet therapy, and was unlikely to have any adverse influence on the course of the underling atherothrombotic disease.

In terms of benefit/risk ratio, therefore, mesoglycan compares favorably with pharmacological therapies for PAOD and represents an attractive option for the medical management claudication [19].

### 2.3. Venous Disease (Table 3)

Some studies suggest the efficacy of mesoglycan and GAG in venous pathology too [23–30].

Chronic venous insufficiency (CVI), the postthrombotic syndrome (PTS), and ulceration represent an important medical problem because of its prevalence (0.3% of the Western population) and healthcare costs [23, 27].

Arosio et al. [23] found a faster and more frequent ulcer healing in patients with chronic venous ulcers treated with mesoglycan in addition to established venous ulcer therapy (limb compression and topical wound care). One hundred and eighty-three patients were randomized to the treatment with mesoglycan (30 mg intramuscularly for three weeks followed by 100 mg/day orally) (*n* = 92) or placebo (*n* = 91) and the treatment was continued until complete ulcer healing or for 24 ± 1 weeks [23]. The estimated time to heal 75% of the patients was 90 days on mesoglycan versus 136 on placebo, while the cumulative rate of healing by the end of observation was 97% versus 82%, respectively, [23]. The difference between treatments was statistically significant (*P* <  .05).

The mechanism by which ulcer healing was promoted by treatment with mesoglycan is not clear. Mesoglycan may counteract putative mechanism for venous ulceration through inhibition of neutrophil adhesion and activation [28], prevention of endothelial barrier function, prevention of fibrin formation, and enhancement of fibrinolysis [23, 28]. An alternative explanation is suggested by recent reports highlighting the role of physiological dermatan and heparin sulphate in wound healing processes [29], including cutaneous wound repair [30].

Andreozzi [24] et al. demonstrated an effectiveness of mesoglycan treatment in preventing thrombotic recurrence in patients with previous DVT, and improvement in disability, pain, and oedema in patients with CVI in a retrospective analysis. The clinical data have been selected from the outpatient database of the Chair of Angiology of the University of Catania (from 1988 to 1997) through a cross survey between the prescription commercial name of mesoglycan and the key words: varicose veins, DVT, CVI, postthrombotic syndrome (PTS), venous thrombosis, and venous ulcer [24]. Patients have been selected on the basis of data relative to principal diagnosis, clinical history, clinical and instrumental objective phlebological picture, dosage and duration of treatment, and follow-up visits in the first three years following the first observation: Group 1 : 56 patients with first episode DVT; Group 2 : 27 patients with recurrent DVT; Group 3 : 182 patients with CVI (107 with primitive CVI and 75 with secondary CVI). DVT patients were evaluated for recurrence prevalence during the follow up period (6, 12, 18, 24, 30, and 36 months). In group 2 the recurrence prevalence in the normal follow up period was evaluated and, in addition, the clinical chronology of the recurrence previous to observation was drawn, in order to find out the recurrence prevalence of the thrombotic episode preceding the study. CVI patients were classified according to CEAP criteria and the efficacy of the treatment was assessed according to the changes in the scores of venous dysfunction (disability score, pain, oedema, skin color change, and cutaneous ulcer). The mean dose of mesoglycan was 50 mg twice daily. The results obtained in groups 1 and 2 showed that mesoglycan was effective in preventing thrombotic recurrence in patients with previous DVT. The recurrence prevalence in patients with DVT at first episode was lower than the prevalence reported by the literature data (17.5% within 2 years and 24.6% within 5 years). The positive trend was also confirmed in the recurrence DVT group, although with a major prevalence (18.51%) due to higher thrombotic risk. Mesoglycan was also effective in CVI patients, with a significant improvement of disability, pain, oedema, and quality of life [24]. This retrospective analysis outlines an interesting therapeutic profile for mesoglycan in chronic venous disorders, and studies are needed in this issue.

The asymmetrical lower extremity swelling is known as “mechanical oedema” (MO) and may interest ankle, calf, and knee. The treatment of MO needs a multidisciplinary approach which includes specific training for muscular pump and pharmacological treatment focused to correct venous endothelium alterations [25, 31–33].

The study of Viliani et al. [25] evaluated the clinical efficacy of mesoglycan 50 mg twice a day in patients affected by MO. Forty-four patients with MO were randomized in two treatment groups: (1) specific physiotherapy (Fkt) alone or (2) Fkt plus mesoglycan 50 mg twice a day, per os. The patients were evaluated before treatment (T0) and after 1 month of treatment (T1), measuring ankle joint range of motion (degrees), calf circumference, and malleolar circumference (cm), pain Borg CR10 scale and adapted lymphoedema Weiss Scale. At final evaluation of the objective and subjective parameters, the mesoglycan effect combined to the Fkt provided statistical differences on nearly all the parameters in comparison with the patients randomized to fkt alone [25]. The present study suggest that mesolgycan treatment (50 mg per os twice a day) can improve the recovery of MO, and it is well tolerated by the patients. Specific physiotherapy remains the first treatment for the recovery of both muscular pump and correct walking, but the optimal treatment of MO seems to be a synergic approach, including both pharmacological and mobilization programs.

Prandoni et al. studied ninety consecutive patients affected by venographically proven DVT of the lower limbs, were given full-dose heparin followed by oral anticoagulants for 12 weeks and then selected randomly to receive, for one year, either mesoglycan (72 mg/day orally) or placebo with a double-blind protocol [26]. All patients wore elastic graduated compression stockings and were prospectively followed for a period ranging from 5 to 48 months. In each scheduled examination programmed every three months for one year and then twice per year, an accurate clinical evaluation was performed and a predetermined objective score was applied. Furthermore, impedance plethysmography and Doppler ultrasound tests were executed serially to assess the persistence of venous obstruction and/or the development of valve incompetence. After a mean follow-up of 3 years, 80% of the patients were totally asymptomatic, and severe postthrombotic sequelae (ulcer and/or oedema) were recorded in only 6 patients (6.6%). The authors failed to identify any correlation between post-thrombotic sequelae and persistence of venous obstruction (as shown by impedance plethysmography) or development of valve incompetence (as shown by Doppler ultrasound test). The behaviour of patients treated with mesoglycan did not differ from that of patients treated with placebo. However, objectively documented recurrences of DVT and/or pulmonary embolism were less frequent in patients treated with mesoglycan (6.6 versus 11.1%, nonsignificant difference), and the only two deaths attributable to pulmonary embolism occurred among the patients treated with placebo [26].

The other GAG preparation, sulodexide, that is administered intramuscularly or orally has shown to increase venous ulcer healing in a placebo-controlled trial of 235 patients without an apparent increase in side effects [34–36].

## 3. Conclusions and Perspectives

Vascular GAG are essential component of the endothelium and vessel wall and are involved in several biologic functions. Mesoglycan is a natural GAG preparation, with antithrombotic and pro-fibrinolytic activities, which has been shown to be clinically effective in a number of vascular atherosclerotic disorders with thrombotic risk. GAG have several actions on the fibrinolytic system, on vascular endothelium, and on rheologic parameters. Mesoglycan has proven efficacy in reducing plasma FBR levels and the risk of recurrences in patients with cerebral arterial disease, with a good safety profile. These antithrombotic and pro-fibrinolytic activities, although not indicated in the treatment of acute arterial or venous thrombosis due to the low anticoagulant effect, suggest that mesoglycan may be useful in the management of vascular diseases, when combined with antithrombotics in the case of disease of cerebral vasculature, and with antithrombotics and vasodilator drugs in the case of chronic peripheral arterial disease. The protective effect of mesoglycan in patients with venous thrombosis or PTS and the absence of side effects support the use of GAG in patients with CVI and persistent venous ulcers, in association with compression therapy (zinc bandages, multiple layer bandages, etc.), elastic compression stockings, and local care, and in the prevention of recurrences in patients with previous DVT, following the standard course of oral anticoagulation treatment.

## Figures and Tables

**Table 1:** Mesoglycan in ischemic stroke. Clinical studies.

Author	Study design	Pathology	Patients (*n*)	Doses/route	Results
Orefice [12]	Prospective	Stroke (1–3 mo before)	30	Mesoglycan 50 mg twice daily orally for 3 months	Mesoglycan: safe and effective in reducing FBR without interfering with other coagulative parameters
Orefice [11]	Randomized Controlled	Stroke (2-3 mo before)	46	Mesoglycan 50 mg twice daily orally versus Ticlopidine 250 mg twice daily, for 2 months	Mesoglycan and ticlopidine: both safe and effective in reducing FBR without interfering with other coagulative parameters
Vecchio [13]	Prospective	Stroke (within 2 mo)	20	Mesoglycan 30 mg twice daily intramuscular, for 15 days	Mesoglycan: significant reduction in FBR, cholesterol, triglycerides, improved erythrocyte filterability without interfering with other coagulative parameters
Mansi [14]	Prospective	TIA or stroke (within 3 mo)	30	Mesoglycan 50 mg twice daily orally	Mesoglycan decreases neurologic deficits
Forconi [15]	Multicenter clinical trial	History of stroke, TIA, RIND or minor stroke	1,398	Mesoglycan 30 mg twice daily i.m., then 100 mg daily orally, versus ASA 300 mg daily	No differences. ASA: higher incidence of side effects

**Table 2:** Mesoglycan in peripheral obstructive arterial disease (POAD). Clinical studies.

Author	Study design	Pathology	Patients (*n*)	Doses/route	Results
Andreozzi [18]	Prospective	POAD in stages I-II Fontaine	10	Mesoglycan 60 mg daily for 20 days	Mesoglycan: safe and effective in improving the wall response to vasodilator stimulus
Raso [19]	Prospective	POAD stage IIb Fontaine	36	Mesoglycan 60 mg daily endovenous for 10 days then 100 mg daily orally for 20 days, repeated for two months then oral mesoglycan for 12 months	Mesoglycan: significant improvement of symptoms and signs in all patients but one
Nenci [20]	Randomized, double-blinded	PAOD stage II Fontaine	242	Mesoglycan 30 mg daily i.m. for 3 weeks then 100 mg daily orally for two weeks, versus matching placebo All patients receive ASA	Mesoglycan: significant clinical improvement versus placebo. Significant improvement in quality of life scores.

**Table 3:** Mesoglycan and venous disease.

Author	Study design	Pathology	Patients (*n*)	Doses/route	Results
Arosio [23]	Randomized	Chronic venous ulcers	183	Mesoglycan 30 mg daily i.m. for 3 weeks then 100 mg daily orally, versus placebo, for 24 ± 1 weeks	Mesoglycan: significant difference in the rate of ulcer healing versus placebo
Andreozzi [24]	Retrospective analysis	Patients with previous DVT	36	Mesoglycan 50 mg twice daily orally	Mesoglycan: effective in preventing thrombotic recurrences
Viliani [25]	Randomized	Mechanical oedema (MO)	44	FKT+Mesoglycan 30 mg twice daily orally versus FKT alone	Mesoglycan: significant clinical improvement in objective and subjective parameters
Prandoni [26]	Randomized	DVT venographically proved	90	Heparin and then oral anticoagulation (12 weeks), then mesoglycan 72 mg daily orally versus placebo, for 1 year	Recurrences of DVT and/or PE less frequent with mesoglycan versus placebo (nonsignificant difference)
